# Protein arginine methyltransferase 1 is required for the maintenance of adult small intestinal and colonic epithelial cell homeostasis

**DOI:** 10.7150/ijbs.89958

**Published:** 2024-01-01

**Authors:** Zhaoyi Peng, Lingyu Bao, Bingyin Shi, Yun-Bo Shi

**Affiliations:** 1 Department of Endocrinology, The First Affiliated Hospital of Xi'an JiaoTong University, No. 277, West Yanta Road, Xi'an, Shaanxi 710061, P.R. China.; 2 Section on Molecular Morphogenesis, Eunice Kennedy Shriver National Institute of Child Health and Human Development, National Institutes of Health, Bethesda, Maryland, MD, USA.

**Keywords:** Epithelial homeostasis, Arginine methylation, methyltransferase, Intestine, Self-renewal, Stem cell

## Abstract

The vertebrate adult intestinal epithelium has a high self-renewal rate driven by intestinal stem cells (ISCs) in the crypts, which play central roles in maintaining intestinal integrity and homeostasis. However, the underlying mechanisms remain elusive. Here we showed that protein arginine methyltransferase 1 (PRMT1), a major arginine methyltransferase that can also function as a transcription co-activator, was highly expressed in the proliferating cells of adult mouse intestinal crypts. Intestinal epithelium-specific knockout of PRMT1, which ablates PRMT1 gene starting during embryogenesis, caused distinct, region-specific effects on small intestine and colon: increasing and decreasing the goblet cell number in the small intestinal and colonic crypts, respectively, leading to elongation of the crypts in small intestine but not colon, while increasing crypt cell proliferation in both regions. We further generated a tamoxifen-inducible intestinal epithelium-specific PRMT1 knockout mouse model and found that tamoxifen-induced knockout of PRMT1 in the adult mice resulted in the same region-specific intestinal phenotypes. Thus, our studies have for the first time revealed that the epigenetic enzyme PRMT1 has distinct, region-specific roles in the maintenance of intestinal epithelial architecture and homeostasis, although PRMT1 may influence intestinal development.

## Introduction

The intestinal epithelium establishes a physical barrier between the external and internal compartments of the body and plays a key role in food digestion and nutrient absorption. These functions, in a constantly self-renewing tissue, rely on a complex and precise control of cell proliferation, differentiation, and death to ensure tissue homeostasis 1, 2. The epithelium of the small intestine and large intestine (colon) consists of crypt/villus and crypt/surface unit 2, respectively. At the base of crypts, adult intestinal stem cells (ISCs) proliferate for self-renewal and/or to generate epithelial progenitor cells, known as transit-amplifying (TA) cells. TA cells rapidly proliferate and further give rise to fully differentiated cells. There are 2 main types of intestinal epithelial cell lineages. One is absorptive enterocytes and the other is secretory cells, including goblet cells (secreting mucins), enteroendocrine cells (secreting hormones), and Paneth cells (secreting antimicrobial peptides). Except for Paneth cells, the differentiated cells move up towards the tip of the villus, which takes 3-5 days in mouse, and eventually undergo apoptosis and are shed into the intestinal lumen. Paneth cells are the only differentiated cells that migrate downwards to the bottom of small intestinal crypt 1-7. Diverse signaling pathways, including Wnt, Notch and BMP signaling pathways, are known to be involved in intestinal morphogenesis during development and maintenance of intestinal homeostasis in adult 1, 8, 9. However, the underlying mechanisms for maintaining homeostasis remain elusive.

Protein arginine methyltransferase 1 (PRMT1) is the predominant arginine methyltransferase in mammalian cells and responsible for over 85% of arginine methylation activity 10. PRMT1 methylates a variety of histone and non-histone protein substrates involved in multiple cellular functions such as transcription, DNA damage response, and cell proliferation 11, 12. PRMT1 total knockout mice are embryonic lethal 13. Conditional knockout studies have revealed the important roles of PRMT1 in different cellular processes. For example, loss of PRMT1 in the central nervous system induces severe hypomyelination and developmental defects 14, and deletion of PRMT1 from vascular smooth and skeletal muscle leads to contractile dysfunction and muscle atrophy, respectively 15, 16. PRMT1 has been considered a key epigenetic regulator of hematopoiesis and mature β-cell identity 17, 18. In addition, PRMT1 is required for maintenance of progenitor cells in mice epidermis, a self-renewing mammalian tissue 19.

We previous discovered that PRMT1 expression is upregulated in the intestine during vertebrate postembryonic development, including intestinal remodeling during *Xenopus* metamorphosis and intestinal maturation during neonatal mouse development, both of which leads to the formation of the adult intestine 20, 21. Moreover, transgenic and knockdown studies indicated an important role of PRMT1 in the formation and/or proliferation of ISCs during *Xenopus* metamorphosis 20. Using the TALEN genome editing technology to knockout PRMT1 in the diploid anuran *Xenopus tropicalis*, we observed that PRMT1 is essential for tadpole growth and development with the knockout tadpoles die before metamorphosis, accompanied by drastic reduction in cell proliferation in tissues like the brain 22. To further study the role of PRMT1 in vertebrate intestinal development and maturation, we developed an intestinal epithelium-specific PRMT1 knockout mouse model and observed that adult PRMT1 knockout mice had defects in the small intestine, with more elongated crypt morphology and surprisingly increased cell proliferation in the crypts 23.

In this study, we further investigated the role of PRMT1 in the mouse intestine. We found that PRMT1 is highly expressed in the mouse crypts of both small intestine and colon, where the proliferating cells and stem cells were located. Intestinal epithelial specific knockout of PRMT1 also led to increased cell proliferation in the colonic crypts, just like in the small intestine. Surprisingly, we found that colonic goblet cells were reduced, in contrast to the increased goblet cell number in the crypts of the small intestine and lack of changes in the total goblet cells in the small intestine 23, revealing a region-specific role of PRMT1 in the mouse intestine. By using an inducible intestinal epithelium-specific PRMT1 knockout mouse model, we observed that knocking out PRMT1 in adult mouse intestinal epithelium caused the same changes in the small intestine and colon, i.e., increased crypt cell proliferation and region-specific changes in goblet cells, as observed with mice with epithelial specific PRMT1 knockout during development. Our findings, thus, demonstrated for the first time that the epigenetic enzyme PRMT1 plays critical region-specific roles for maintaining adult intestinal epithelial cell architecture and homeostasis.

## Results

### Intestinal epithelium-specific knockout of PRMT1 (PRMT1^ΔIEC^) increases cell proliferation and reduces goblet cells in the adult colon

**Figure 1:**
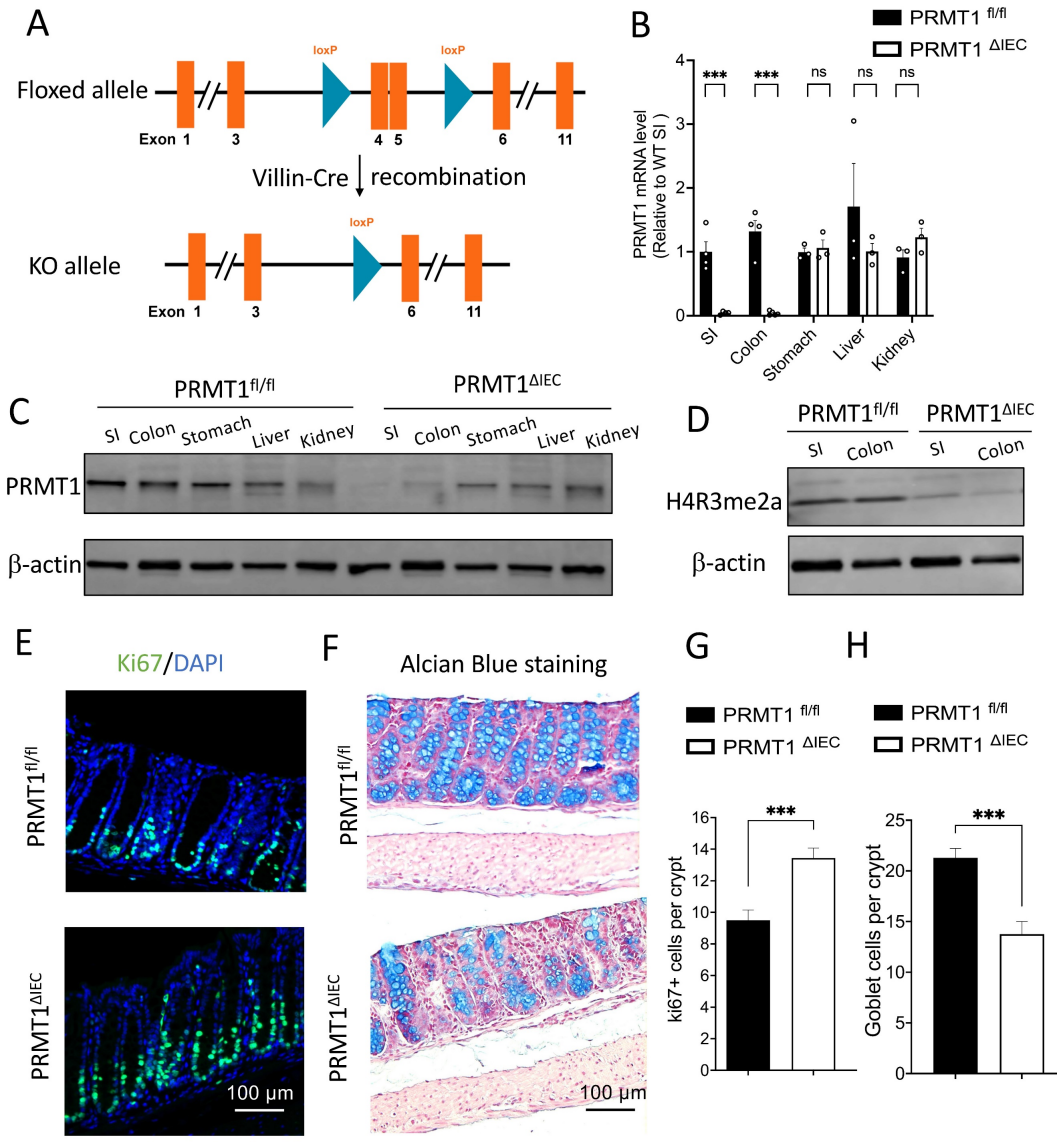
Intestinal epithelium-specific knockout of PRMT1 leads to increased cell proliferation and a reduction in goblet cells in the colon. (A): Experimental scheme to generate intestinal epithelium-specific PRMT1 knockout (PRMT1^ΔIEC^). Mice with two loxP sites flanking exons 4 and 5 (PRMT1^fl/fl^) were crossed with mice expressing Cre recombinase under the control of the villin promoter (Villin-Cre), causing recombination via the LoxP sites to delete exons 4 and 5 24, 49 (https://www.mousephenotype.org/data/genes/MGI:107846#order. Note that in our earlier paper 23, the floxed exons were referred to as exons 5 and 6 due to the use of a different PRMT1 RNA isoform with 12 total exons instead of the 11 exon RNA isoform used here). (B, C): The mRNA and protein levels of PRMT1 in different organs of PRMT1^ΔIEC^ and control (PRMT1^fl/fl^) littermates as determined by RT-qPCR (B) and Western blot analyses (C), each dot represents an individual mouse. The values were presented as mean ± SEM with n=3-4 mice per group. ***p < 0.001. β-actin was used as a loading control. SI: small intestine. Note the loss of PRMT1 specifically in the intestine but not other organs of PRMT1^ΔIEC^ mice. (D): Western blot analyses of H4R3me2a protein in the small intestinal (SI) and colonic epithelium of PRMT1^ΔIEC^ and control mice with β-actin as a loading control. Note the reduction in methylation of H4R3 (H4R3me2a level) in the intestine of PRMT1^ΔIEC^ mice. (E): Representative images of Ki67 immunofluorescent staining (green) of colonic sections from PRMT1^ΔIEC^ and control littermates. The DNA was stained with DAPI (blue). (F): Representative images of Alcian Blue staining of colonic sections from PRMT1^ΔIEC^ and control mice. Goblet cells were stained in blue. (G, H): Quantification of E and F showed that PRMT1 deletion increased cell proliferation (G) and reduced the number of goblet cells in the colon (H). Multiple sections per animal were analyzed for each group. The values were presented as mean ± SEM with n=3-4 mice per group at each time point. ***p < 0.001. ns no significant. Scale bars indicate 100 μm.

To study the role of PRMT1 in intestinal development and maturation, we previously generated a tissue-specific PRMT1 knockout mouse model by crossing a PRMT1 floxed (PRMT1^fl/fl^) mouse line with mouse line expressing the Cre recombinase under the control of the villin promoter for intestinal epithelial specific expression of Cre (Vil-Cre) (Fig. 1A) 23. The Cre-mediated recombination of the floxed PRMT1 gene resulted in PRMT1 knockout (deleting the floxed exons 4 and 5) specifically in the intestinal epithelium during embryogenesis 24 (PRMT1 ^fl/fl^; Vil-Cre, henceforth referred to as PRMT1^ΔIEC^) (Fig. 1A). We observed that the PRMT1 deletion increased cell proliferation in the crypts and altered cell differentiation in adult mouse small intestine 23. To further investigate the function of epithelial PRMT1 in intestinal development and homeostasis, we compared the colon (large intestine) of adult wild type (control or PRMT1^fl/fl^) and PRMT1 knockout (PRMT1^ΔIEC^) mice (Fig. 1A). We first validated the efficient deletion of PRMT1 at both mRNA and protein level by RT-qPCR and Western blot analyses (Fig. 1B, C). The results showed that PRMT1 mRNA and protein levels were very low or non-detectable in PRMT1^ΔIEC^ small intestinal and colonic epithelium, but were similar in several other organ (stomach, liver and kidney) between the wild type and knockout mice (Fig. 1B, C), demonstrating the efficiency and tissue specificity of the knockout. PRMT1 is a major arginine 3 of histone H4 (H4R3) methyltransferase 25, 26. Consistently, we observed PRMT1 deficiency dramatically decreased global levels of asymmetrical dimethylation on H4R3 (H4R3me2a) in both small intestinal and colonic epithelia (Fig. 1D).

The PRMT1^ΔIEC^ colon had normal gross morphology, additionally, there was no difference in the length of the colon between wild type and mutant mice (data not shown). We next analyzed the colonic sections of adult PRMT1^ΔIEC^ mice and wild type mice. In wild type mice, Ki67-labeling showed that Ki67+ proliferating cells were expectedly abundant near the bottom of the colonic crypts and were reduced or absent in epithelial cells further up toward the top of the crypts (Fig. 1E). PRMT1 knockout increased ki67+ proliferating cells (Fig. 1E, G), similar to what observed in the small intestine 23. Interestingly, Alcian Blue staining for goblet cells, the second most abundant differentiated epithelial cells in the colon, showed that the number of goblet cells was decreased in colonic crypts of PRMT1^ΔIEC^ mice (Fig. 1F, H), in contrast an increase observed in the crypts of the small intestine of PRMT1^ΔIEC^ mice (although overall goblet cell number in the small intestine was not affected by PRMT1 knockout) 23. These findings suggested an important and region-specific role of PRMT1 in the development and/or homeostasis of the intestine.

### PRMT1 is highly expressed in the mouse intestinal crypts of the small intestine and colon

**Figure 2:**
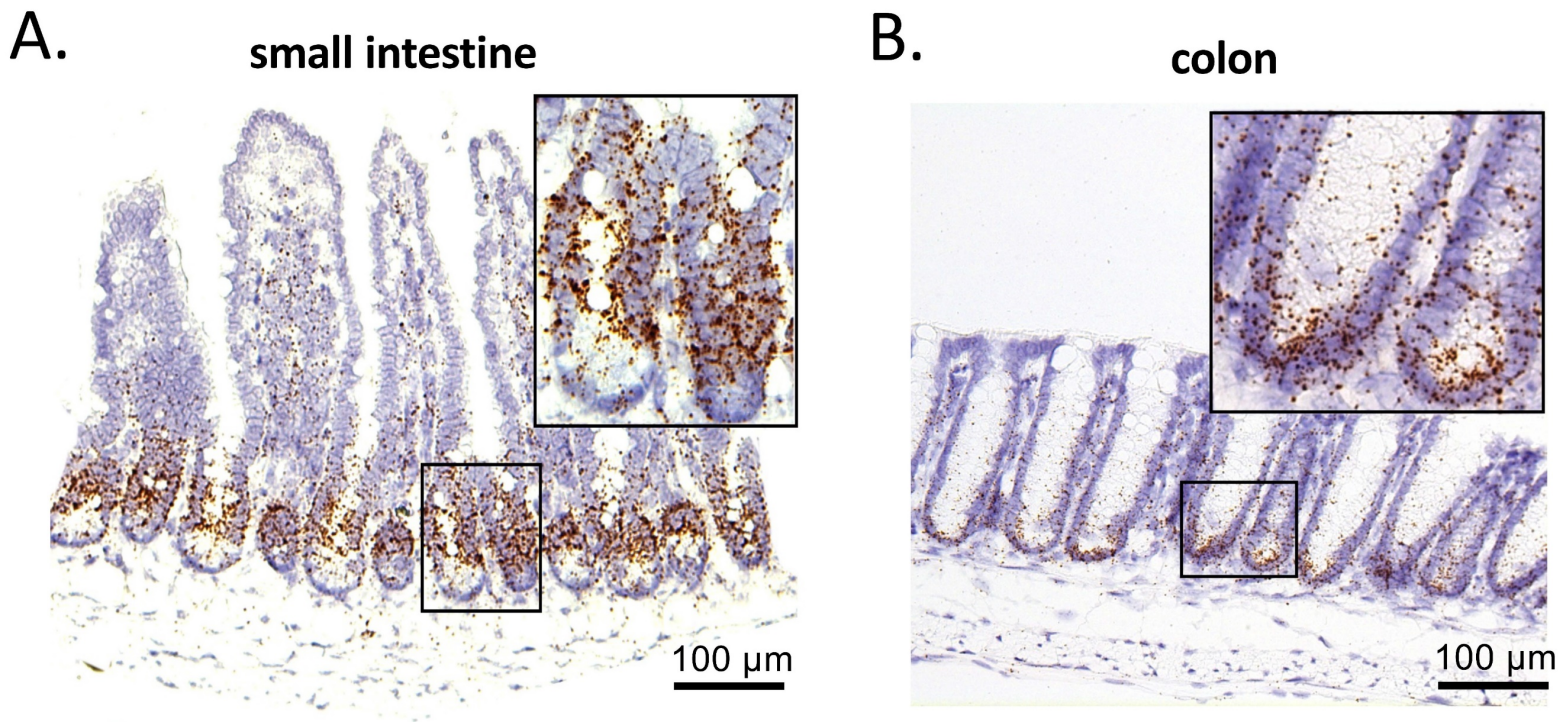
PRMT1 is highly expressed in the crypts of small intestine and colon. PRMT1 expression was analyzed by in situ hybridization on small intestinal (A) and colonic (B) sections of adult mice, with the inset showing the boxed area. Scale bars indicate 100 μm.

We have previously found that high levels of PRMT1 mRNA are expressed in an evolutionally conserved manner during the formation of the adult intestine in vertebrates 20. To determine PRMT1 expression in the adult mouse intestine, we performed in situ hybridization on paraffin-embedded sections of the adult small intestine and colon of wild type mice. In the small intestine, PRMT1 mRNA was highly expressed in the transit-amplifying (TA) zone of the crypts, but not in villi (Fig. 2A), in consistent with earlier immunohistochemical analysis of PRMT1 protein 23. Similarly, in the colon, PRMT1 mRNA was highly expressed in near the bottom of the crypts (Fig. 2B), colocalizing with the proliferating cells (Fig. 1E). These expression patterns suggest that PRMT1 regulates cell proliferation directly in both the small intestine and colon to help maintain epithelial homeostasis in the adult intestine.

### PRMT1 is require for the maintenance of adult intestinal morphology

**Figure 3:**
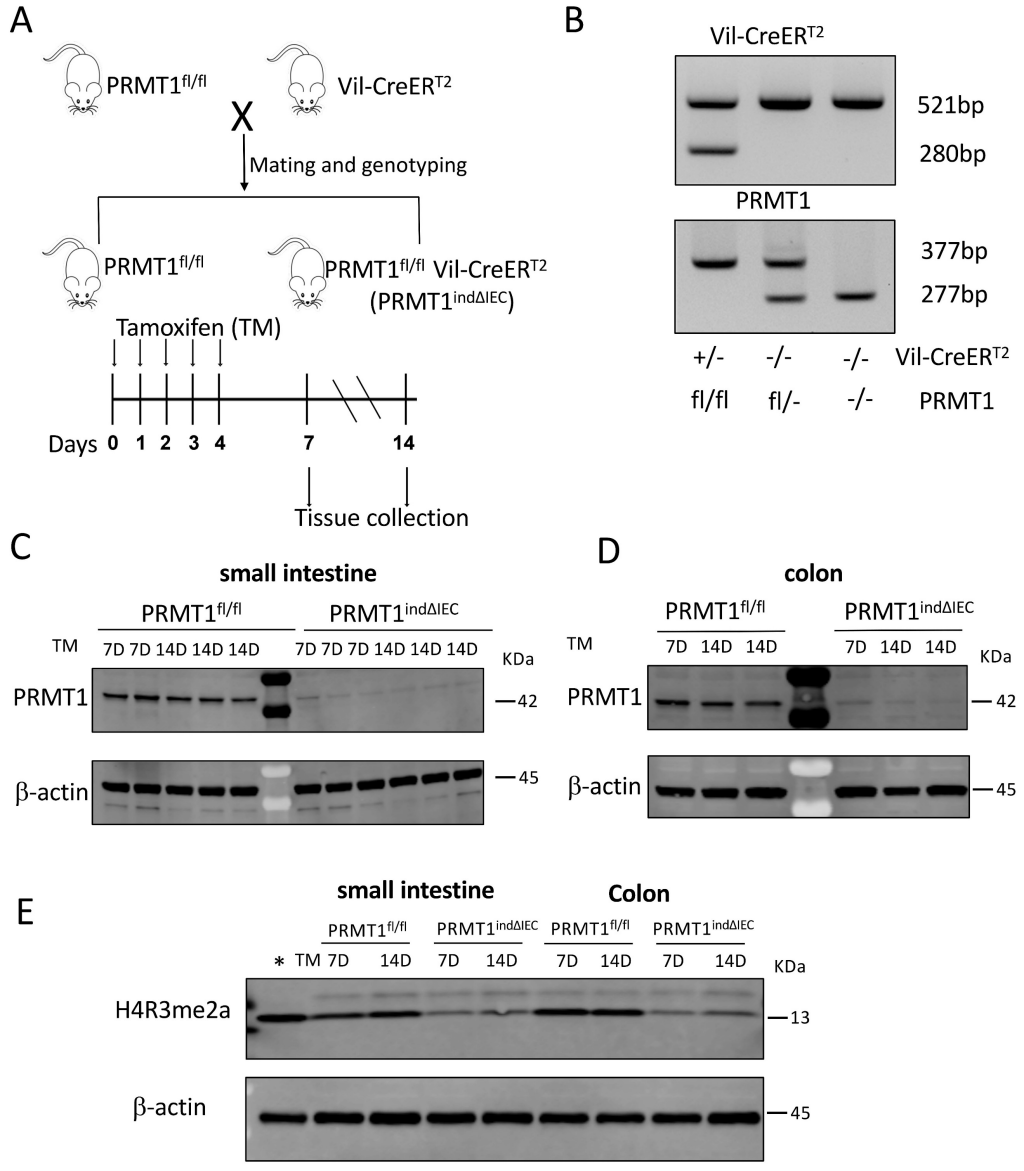
Generation of inducible conditional PRMT1 knockout mice by crossing PRMT1^fl/fl^ mice with Vil-CreER^T2^ mice, which expresses tamoxifen (TM)-dependent Cre (CreER^T2^). (A): Schematic diagram for inducible PRMT1 deletion in adult intestinal epithelium with TM treatment. Upper: generation of PRMT1^fl/fl^ and PRMT1^fl/fl^ Vil-CreER^T2^ (PRMT1^indΔIEC^) mice; Lower: TM injection and tissue collection. The PRMT1^fl/fl^ and PRMT1^indΔIEC^ mice were injected with 75mg/kg TM for 5 consecutive days (day 0-4). Intestinal tissues were collected at the indicated days. (B): PCR-genotyping of genomic DNA extracted from tail biopsies. The tail biopsies were collected at the time of weaning (around day 21 after birth). Upper panel: genotyping with CreER^T2^ primers. The 521-bp PCR product was detected in wild-type mice while both 521 and 280 bp PCR products were present in CreER^T2^ mice. Lower panel: genotyping with PRMT1 primers. The 277-bp PCR product was detected in wild-type mice while the 377-bp PCR product was present in PRMT1^fl/fl^ mice. In PRMT1^fl/-^ mice, both 377 and 277 bp PCR products were detected. (C, D): Western blot analyses of PRMT1 protein in the small intestinal (C) and colonic (D) epithelium of the indicated mouse strains on 7 and 14 after the first TM injection, with β-actin as a loading control. Each lane was loaded with equal amount of the lysate obtained from an individual mouse. (E): Western blot analyses of H4R3me2a protein in the small intestinal and colonic epithelium of the indicated mouse strains 7 and 14 days after the first TM injection, with β-actin as a loading control. The first lane on the left (marked with *) contained a protein sample from whole *Xenopus tropicalis* tadpoles and lacked the upper band in the H4R3me2a panel, suggesting that the band in mouse samples was non-specific.

To determine if PRMT1 is indeed critical for adult intestinal homeostasis, we generated an inducible conditional PRMT1 knockout mouse line by crossing PRMT1^fl/fl^ mice with Vil-CreER^T2^ mice, which expresses tamoxifen (TM)-dependent Cre recombinase (CreER^T2^) in the intestinal epithelium (Fig. 3A, B). The resulting PRMT1^indΔIEC^ (PRMT1^fl/fl^ Vil-CreER^T2^) mice would have normal PRMT1 expression throughout development in the absence of tamoxifen. Upon injection of tamoxifen into PRMT1^indΔIEC^ mice, CreERT2 becomes active, leading to the conditional knockout of the PRMT1 gene specifically in the intestinal epithelium. As expected, tamoxifen treatment of adult PRMT1^indΔIEC^ but not the control PRMT1^fl/fl^ mice resulted in the loss of PRMT1 protein in the epithelium of both small intestine and colon of all injected mice on 7 or 14 days after the first tamoxifen injection (Fig. 3C, D). In addition, Western blot analysis showed that tamoxifen-induced loss of PRMT1 also led to a significant reduction in H4R3me2a levels in the epithelium of both small intestine and colon (Fig. 3E).

**Figure 4:**
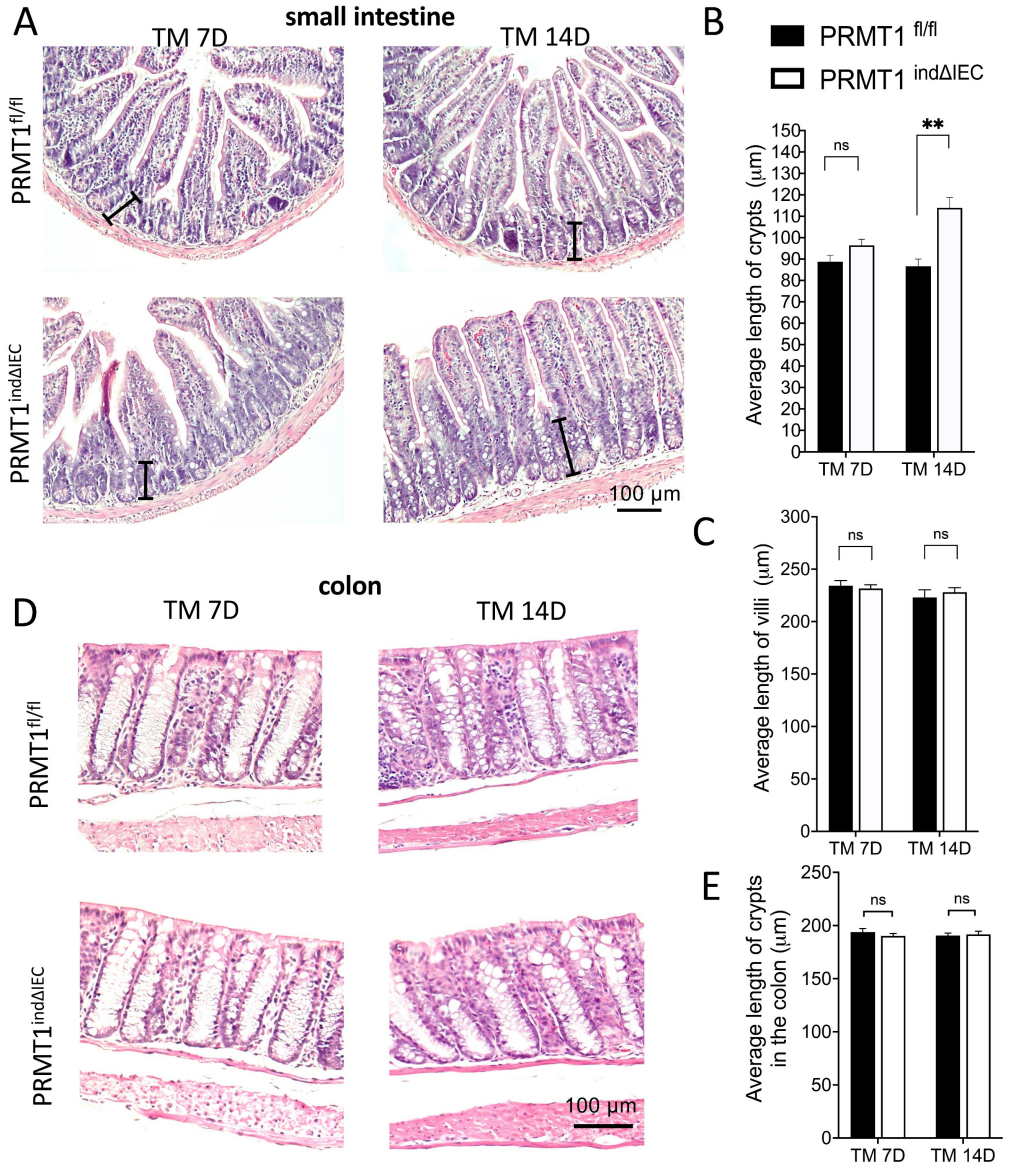
Inducible deletion of PRMT1 in the intestinal epithelium leads to elongated crypts in small intestine on Day 14 after the initiation of tamoxifen treatment. (A, D): Representative images of H&E staining of small intestinal (A) and colonic (D) sections from PRMT1^indΔIEC^ and control littermates at indicated days of TM treatment as described in Fig. 3A. The black lines in (A) indicate individual crypts in small intestine. (B, C, E): The lengths of the crypt (B) and villus (C) in small intestine and crypts (E) in colon were measured from multiple sections per animal by using image J. The values were presented as mean ± SEM with n=3-4 mice per group at each time point. **p < 0.01. ns no significant. Scale bars indicate 100 μm.

Analyses of the small and large intestine (colon) of the tamoxifen-treated adult PRMT1^indΔIEC^ and PRMT1^fl/fl^ mice revealed that knockout of PRMT1 in adult mice did not affect the gross morphology and lengths of the small intestine and colon (data not shown). H&E-stained histological sections of the small intestine showed that mutant intestine had significantly longer crypts 14 days after the initiation of tamoxifen treatment (Fig. 4A, B), whereas the length of villi was not affected by the knockout (Fig. 4A, C). In addition, there was no difference in the length of crypts of the colon between wild type and mutant mice (Fig. 4D, E). These findings are similar to the observations for the adult PRMT1^ΔIEC^ mice (see above and 23), where PRMT1 was knocked out in the intestinal epithelium during development.

### PRMT1 deletion in adult intestinal epithelium increases cell proliferation in the crypts

**Figure 5:**
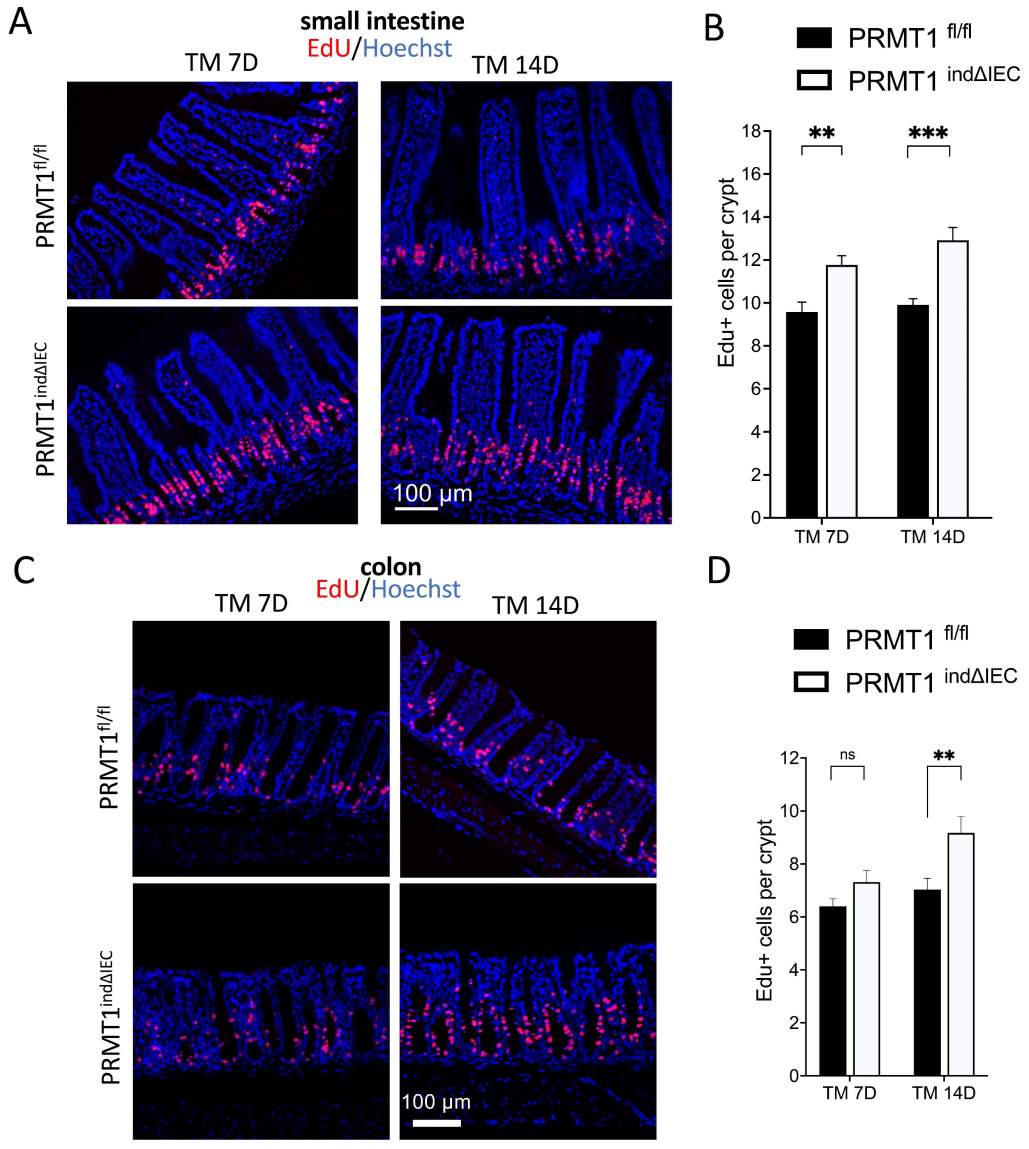
Inducible PRMT1 deletion in adult intestinal epithelium increases cell proliferation in the crypts. (A, C): Representative images of EdU-labeling in small intestinal (A) and colonic (C) sections from PRMT1^indΔIEC^ and control littermates at indicated days after the onset of TM treatment as in Fig. 3A. EdU labeled proliferating cells (red), and the DNA was stained blue with Hoechst. (B, D): Quantification of EdU-positive cells showed that PRMT1 deletion significantly increased cell proliferation in the crypts of small intestine by day 7 (B) and the crypts of colon by day 14 (D) after the initiation of tamoxifen treatment. Multiple sections per animal were analyzed for each group. The values were presented as mean ± SEM with n=3-4 mice per group at each time point. **p < 0.01, ***p < 0.001. ns no significant. Scale bars indicate 100 μm.

We next analyzed cell proliferation in the small intestine and colon by injecting EdU into mice 2 h before sacrificing the mice. EdU is a nucleoside analog incorporated into the DNA during the S-phase. As shown in Fig. 5A, EdU+ cells were mainly located in the middle of the crypts in the small intestine of wild type mice, where the transit-amplifying cells are located. A significant increase in the number of EdU+ cells in the crypts of PRMT1^indΔIEC^ mice was observed on both day 7 and 14 after the initiation of tamoxifen treatment (Fig. 5A, B). The number of EdU+ cells in the crypt of the colon was also increased in the PRMT1^indΔIEC^ mice, although significantly only on day 14 after the initiation of tamoxifen treatment (Fig. 5C, D). To further confirm results, we did immunofluorescence-labeling of the cell proliferation marker Ki67 on small intestine and colon sections and the results were consistent with EdU staining (data not shown). The delay in the increase in colonic epithelial proliferation due to conditional PRMT1 knockout was likely related to a slower epithelium turnover in the colon compared to that in the small intestine 27, 28. Therefore, further analyses on the colon were focused on day 14 after the initiation of tamoxifen treatment.

To determine if the ISC compartment was affected by conditional PRMT1 knockout in the adult mice, we performed in situ hybridization for lgr5, a well-established marker for ISCs 29, 30, on sections of the small intestine and colon of PRMT1^indΔIEC^ and wild type mice after tamoxifen treatment. As expected, lgr5 was detected in the crypt base cells of the small intestine and colon (Fig. S1A, C). There was no difference in the numbers of lgr5+ cells in either small intestine or colon between wild type and PRMT1^indΔIEC^ mice (Fig. S1). Thus, the loss of PRMT1 in adult intestine increases cell proliferation in the crypts without affecting the number of ISCs.

### Tamoxifen-induced deletion of PRMT1 increases cell death in the adult small intestine

**Figure 6:**
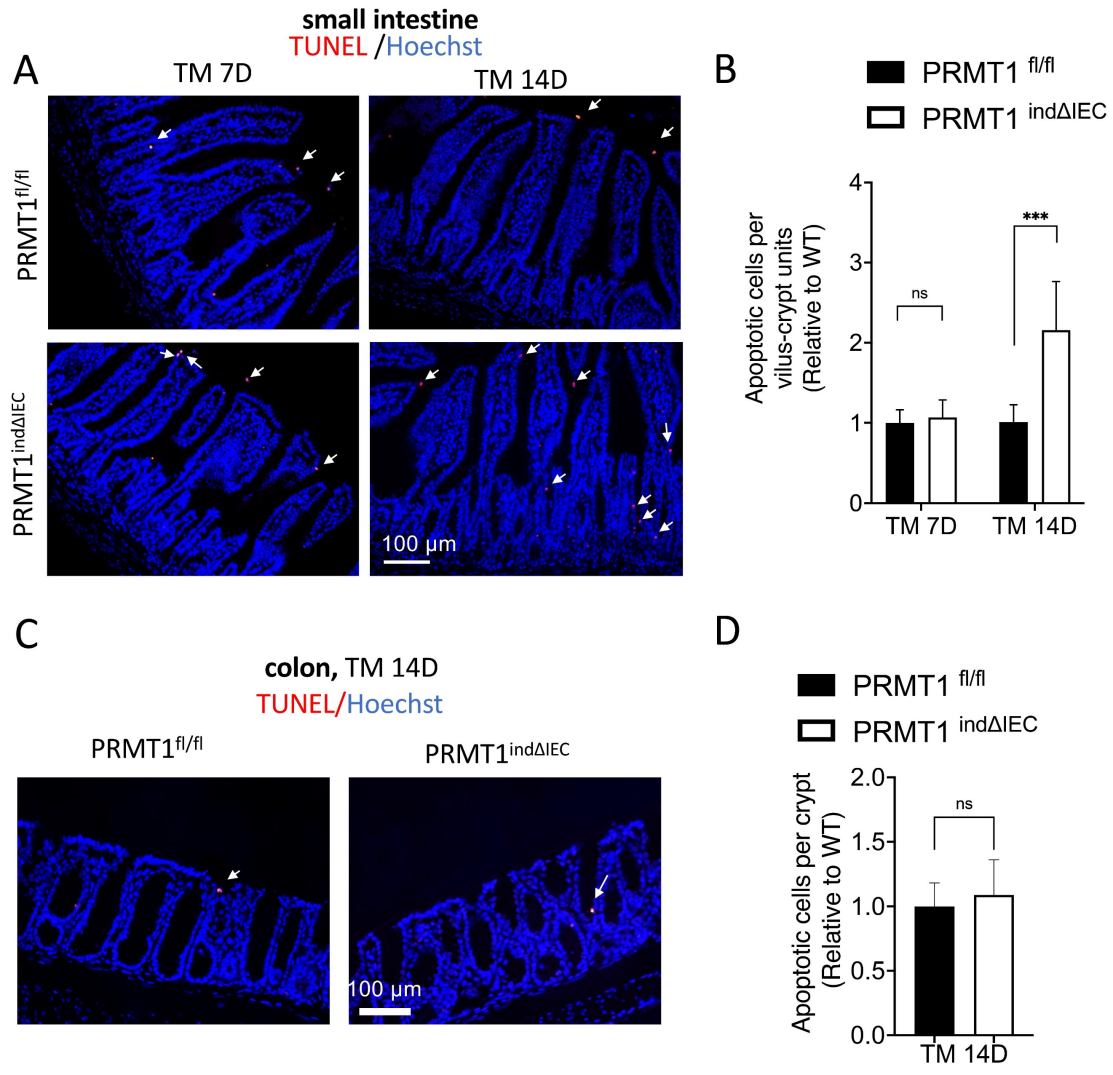
Inducible deletion of PRMT1 in the intestinal epithelium increases cell death in the small intestine. (A, C): Representative TUNEL (terminal deoxynucleotidyl transferase-mediated dUTP nick end labeling) assay images of small intestinal (A) and colonic (C) sections from PRMT1^indΔIEC^ and control littermates at indicated days after the initiation of tamoxifen treatment as in Fig. 3A. White arrows indicate apoptotic cells, which were labeled red with TUNEL assay, and the DNA was stained blue with Hoechst. (B, D): Quantification of TUNEL+ cells of small intestinal (B) and colonic (D) epithelium from PRMT1^indΔIEC^ and control littermates. Multiple sections per animal were analyzed for each group and with the value for the control littermates normalized to 1. The values were presented as mean ± SEM with n=3-4 mice per group at each time point. ***p < 0.001. ns no significant. Scale bars indicate 100 μm.

The adult epithelium undergoes rapid self-renewal with a coordination of cell proliferation and apoptosis in the small intestine 1-3. We observed that the intestine of adult PRMT1^indΔIEC^ mice were morphologically normal except the longer crypts in the small intestine despite the increased cell proliferation (Fig. 4, 5). Thus, we sought to determine if there was a compensatory change in cell death to maintain epithelial homeostasis. We analyzed apoptotic cells by using TUNEL (terminal deoxynucleotidyl transferase-mediated dUTP nick end labeling) assay on small intestine and colon sections of mice after tamoxifen treatment. As shown in Fig. 6A, TUNEL+ cells were mainly located at or near the tips of villi and the number of apoptotic cells was significantly increased on day 14 in the small intestine of tamoxifen-treated PRMT1^indΔIEC^ mice compared to wild type mice (Fig. 6B). In the colonic epithelium, TUNEL+ cells were rare and no significant difference was found between mutant and wild type mice (Fig. 6C, D). Thus, while the rarity of the cell death in the colon made it difficult to quantify possible effect of PRMT1 knockout on cell death in adult colon, our results suggested that the increased cell proliferation and cell death may compensate each other to contribute to the maintenance of the largely normal intestinal structure and homeostasis in the crypts of the PRMT1^indΔIEC^ mice, consistent with a model of epithelial homeostasis through compensatory changes in cell proliferation and cell death 31.

### Conditional deletion of PRMT1 in adult intestinal epithelium alters cell differentiation in the crypts

**Figure 7:**
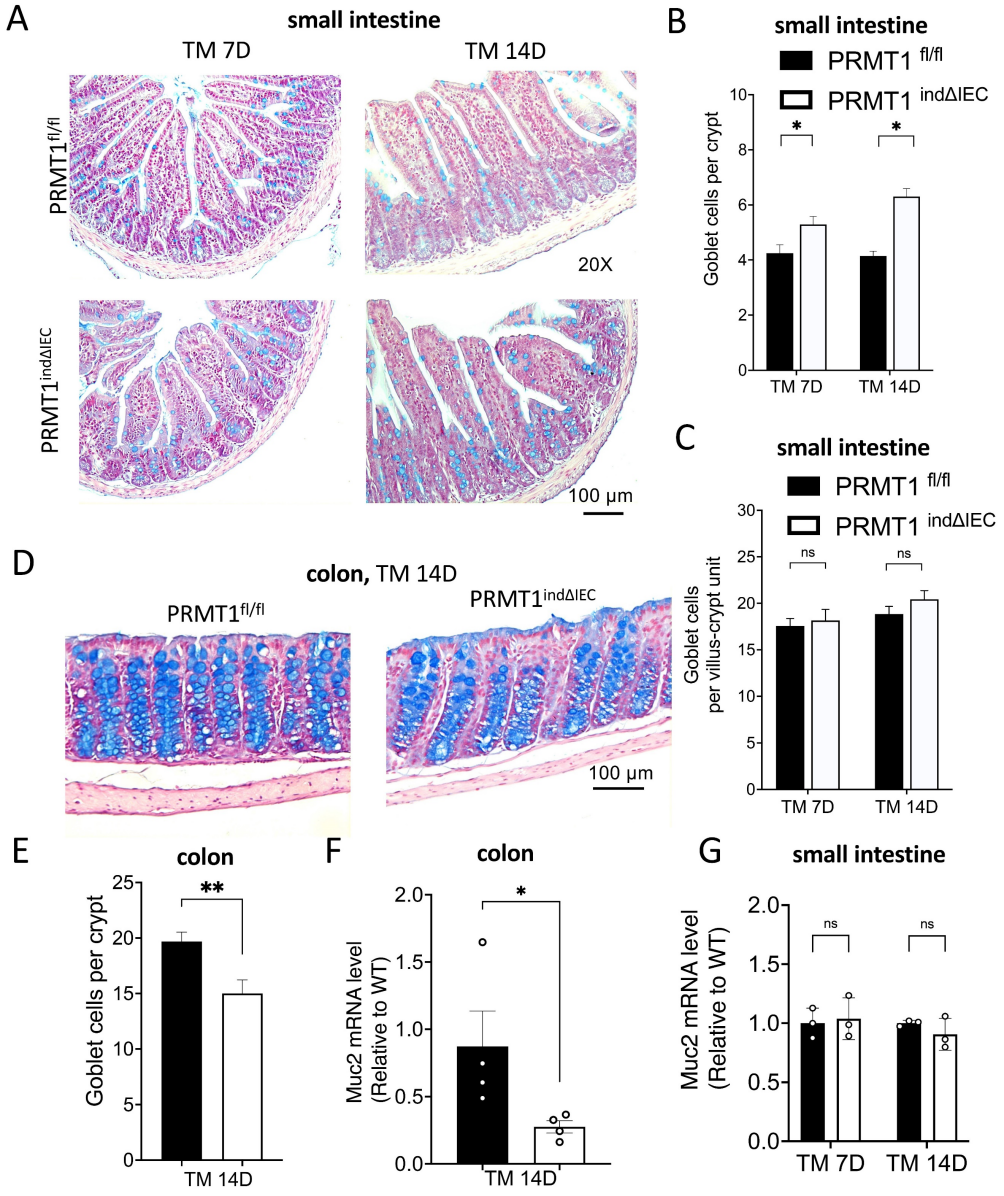
Inducible deletion of PRMT1 in the intestinal epithelium has region-specific effects on goblet cells. (A, D) Representative images of Alcian Blue staining of small intestinal (A) and colonic (D) sections from PRMT1^indΔIEC^ and control littermates at indicated days after the initiation of tamoxifen treatment as in Fig. 3A. Goblet cells were stained in blue. (B, C, E): Quantification shows that PRMT1 deletion increased the number of goblet cells in the crypts of small intestine (B) but reduced it in the crypts of colon (E). Interestingly, in small intestine, the total number of goblet cells per villus-crypt unit did not change (measured by counting the total number of goblet cells in the small intestinal sections and then dividing the total by the number of villi present in the sections) (C). (F, G): Analyses of the expression of Muc2, a marker for goblet cells, in the colonic (F) and small intestinal (G) epithelial cells of PRMT1^indΔIEC^ and control mice at indicated days after the initiation of tamoxifen treatment. Each dot represents an individual mouse. Note that changes in Muc2 expression were in agreement with the changes in the number of goblet cells in the small intestine (C vs. G) and colon (E vs. F), respectively. The values were presented as mean ± SEM with n=3-4 mice per group at each time point. *p < 0.05, **p < 0.01. ns no significant. Scale bars indicate 100 μm.

There are two major differentiated cells in the small intestinal crypts: Paneth cells and goblet cells, whereas the colon lacks Paneth cells. To investigate the effects of PRMT1 deletion in the adult intestinal epithelium on these differentiated cells in adult mice, we first analyzed goblet cells by using Alcian blue staining. As shown in Fig. 7A, PRMT1^indΔIEC^ mice had more goblets cells in the crypts of the small intestine compared to wild type mice on both day 7 and day 14 after the initiation of tamoxifen treatment. However, the total number of goblet cells in the small intestine (i.e., goblet cells per villus-crypt unit) remained unchanged (Fig. 7C). Consistently, the expression of goblet cell marker Muc2 in small intestinal epithelium was similar between the mutant and wild type mice (Fig. 7G). These findings were the similar to the observations in mice with PRMT1 deleted in the intestinal epithelium during development 23. In contrast, the number of goblet cells was significantly decreased in the colon of PRMT1^indΔIEC^ mice after tamoxifen treatment compared to the wild type mice (Fig. 7D, E). Furthermore, the mRNA level of Muc2 was also decreased in the mutant colonic epithelial cells (Fig. 7F). These were again similar to the findings in mice with PRMT1 deleted in the intestinal epithelium during development (Fig. 1F, H). Collectively, our results indicated that PRMT1 deletion had region-specific effects on goblet cell differentiation and/or homeostasis in the adult intestine.

We also performed immunostaining for lysozyme, a well-established marker of Paneth cells (Fig. S2A). Quantitative analyses revealed that the number of Paneth cells in the small intestine was similar between mutant and wild type mice on both day 7 and day 14 after the initiation of tamoxifen treatment (Fig. S2B). We previously showed PRMT1 mice with PRMT1 deletion in the epithelium during development (PRMT1^ΔIEC^) had a significant reduction of Paneth cells in the small intestine 23. This apparent discrepancy was likely due to the fact that Paneth cells have a long lifespan of approximately 1-2 months 32, 33. Therefore, the short-term deletion of PRMT1 in adult mice in our experiments would not be expected to cause significant changes in Paneth cell numbers.

## Discussion

Due to the high turnover and hierarchical architecture of the intestinal epithelium, ISCs have become an adult stem cell paradigm. Cell fate specification and differentiation of adult stem cells rely on the interplays between specific transcription factors and distinct epigenetic landscapes. Whereas many transcription factors involved in stem cell maintenance and differentiation have been defined 3, 34-36, much less is known about epigenetic contributions. Here, our studies indicate that PRMT1, a predominant histone arginine methyltransferase, is required for the maintenance of adult small intestinal and colonic epithelial cell homeostasis.

We first revealed a role of PRMT1 in colonic epithelial homeostasis. We previously showed that PRMT1 altered the structure and epithelial homeostasis in the small intestine by using intestinal epithelium-specific knockout PRMT1 mouse model (PRMT1^ΔIEC^) 23. Many studies have revealed significant phenotypic differences between the small intestine and colon in various mutant mice 34, 37, 38, prompting us to investigate if PRMT1 has different roles in the small intestine and colon. While like in the small intestine, cell proliferation was increased in the colonic crypts of PRMT1^ΔIEC^ mice, the PRMT1^ΔIEC^ colon did not exhibit elongated crypt morphology compared to the wild type mice. Surprisingly, the number of goblet cells was decreased in PRMT1^ΔIEC^ colon (Fig.1D, F), in contrast to the increase in the number of goblet cells in the crypts of PRMT1^ΔIEC^ small intestine. Thus, PRMT1 has region-specific role on goblet cell differentiation and/or maintenance. Goblet cells are known for their role in providing the protective mucus barrier that covers the intestine and their variation in different regions of the intestine may be important for the region-specific needs of the epithelium to respond to local stimuli and environmental changes 39.

As PRMT1 knockout takes place early during development in the PRMT1^ΔIEC^ mice, the intestinal phenotypes in the adult mice could be due to disruption of normal intestinal development and/or maintenance of adult intestinal homeostasis caused by deletion of PRMT1 in the intestinal epithelium. By using a tamoxifen-inducible mouse model that effectively knocks out PRMT1 in the intestinal epithelium within days after injection of tamoxifen into adult mice, we showed conditional knockout of PRMT1 in adult mice also led to increased cell proliferation in the crypts of both small intestine and colon, elongated crypts in the small intestine, as well as region-specific changes in goblet cells in the crypts. These phenotypes are similar to those observed in PRMT1^ΔIEC^ mice, where PRMT1 is knocked out in the intestinal epithelium during embryogenesis. Thus, the intestinal phenotypes due to intestinal epithelial PRMT1 knockout are due to critical roles of PRMT1 in the maintenance of adult small intestinal and colonic epithelial homeostasis, although PRMT1 may also play a role during intestinal development.

PRMT1 knockout also increases intestinal cell death to compensate for the increased cell proliferation to maintain intestinal homeostasis. As PRMT1 is highly expressed only in the crypt, the effect of the PRMT1 knockout on cell death is likely indirect. One possibility is as proposed in a model of epithelial homeostasis through compensatory changes in cell proliferation and cell death 31, that is, increased cell proliferation in the crypt leads to increased cell migration along the villus-crypt axis, thus altering cell-cell and cell-extracellular matrix interactions, which in turn affects cell fate 31.

There have been other studies showing a role of PRMT1 in stem cell maintenance and/or proliferation. PRMT1 was reported to be a critical regulator of hematopoietic stem cell fate and a key mediator of their self-renewal 17. Absence of PRMT1 in muscle stem cells enhances cell proliferation but represses myogenic differentiation after injury 40 and PRMT1 deletion in the murine epidermis leads to impaired progenitor function 19. Our own previous study showed that PRMT1 was upregulated and required for the formation and/or proliferation of the adult intestine during metamorphosis in the anuran *Xenopus laevis,* and that knocking down PRMT1 reduces adult stem cell proliferation during *Xenopus* intestine metamorphosis 20, 21. In addition, high levels of PRMT1 expression were also found during adult intestinal development/maturation in mouse and zebrafish 20. Thus, we would expect that PRMT1 knockout might inhibit intestinal maturation and cell proliferation in mouse. Surprisingly, we found that deletion of PRMT1 in adult intestinal epithelium led to increased cell proliferation. Furthermore, PRMT1 knockout did not affect the stem cells at the base of the crypt. These findings suggest that PRMT1 may have distinct roles in development and adult tissue maintenance/homeostasis in the mouse intestine.

How PRMT1 is involved in intestinal homeostasis remains to be determined. PRMT1 can directly methylate R3 residue of histone H4 to enhance transcription via the formation of transcriptionally active chromatin 25, 26. Consistently, we observed a significant reduction of H4R3me2a level in PRMT1-deficent intestinal epithelium. In fact, we initially studied PRMT1 because its expression is upregulated in the intestine during thyroid hormone-dependent *Xenopus* metamorphosis 20, 21. Our earlier studies suggest that PRMT1 enhances adult ISC formation and/or proliferation by functioning as a co-activator for thyroid hormone receptor during *Xenopus* metamorphosis 20, 21. The surprising finding that PRMT1 knockout in adult mouse intestinal epithelium increases cell proliferation argues that PRMT1 affects adult mouse intestinal homeostasis via a different mechanism not involving thyroid hormone receptor in the proliferating cells in the intestinal crypts directly.

PRMT1 is also known to methylate other proteins to affect different cellular processes. One of the best studied is the Wnt pathway. Accumulating evidence suggests that Wnt signaling pathway is essential for the ISC proliferation in the crypt compartment 41-44. Pinto et al. reported that transgenic mice ectopically expressing DKK1, a secreted Wnt inhibitor, had reduced epithelial proliferation, accompanied by the loss of crypts 43. Similarly, inducible deletion of β-catenin in the intestinal epithelium resulted in the loss of transient amplifying cells and crypt structures 44. More importantly, PRMT1-induced methylation of Axin, a negative regulator of the Wnt pathway, enhanced its interaction with GSK3β, leading to inhibition of the Wnt pathway 45, which would be consistent with our finding here. In addition, Zhao et al. found that genetic ablation of PRMT1 in hepatocytes resulted in up-regulation of β-catenin 46. Thus, it is possible PRMT1 deletion in the adult intestinal epithelium may increase cell proliferation via activation of Wnt/β-catenin pathway. On the other hand, PRMT1 is required for GSK3 sequestration in endolysosomes, a key event in Wnt signaling 47, which would suggest that PRMT1 knockout would reduce Wnt signaling. Furthermore, when we analyzed β-catenin expression in the small intestinal epithelium by Western blot and immunohistochemistry, we failed to find any significant difference between PRMT1^ΔIEC^ and wild type mice (data not shown). Additionally, the expression level of Wnt target genes (Axin2, Ctnnb1, Lef1, MMP7) were also similar in PRMT1^ΔIEC^ and wild type mice (data not shown). Thus, it is likely that PRMT1 may affect cell proliferation through mechanisms other than directly regulating Wnt pathway in the proliferating cells. Clearly, further studies are needed to determine how PRMT1 affect adult intestinal homeostasis and if PRMT1 affects crypt cell proliferation cell-autonomously or indirectly through its function in other epithelial cells, e.g., through the reduction in Paneth cells and goblet cells in the crypts of the small intestine and colon, respectively.

## Materials and methods

### Animals

Mice carrying a LoxP-flanked PRMT1 allele (PRMT1^fl/fl^) were generated as previously reported 23; intestinal epithelium-specific (IEC) knockout PRMT1 mice were generated by crossing PRMT1^fl/fl^ mice with transgenic mice carrying either a constitutively active Cre recombinase under the control of the villin promoter (Vil-Cre) (The Jackson Lab), expressed during embryonic development (from embryonic day 9) and throughout adulthood in intestinal epithelial cells 48, or tamoxifen-inducible Cre under the same promoter (Vil-CreERT2) (The Jackson Lab). For induction of PRMT1 knockout in the CreERT2 line (PRMT1^fl/fl^; vil-Cre^ERT2^, henceforth referred to as PRMT1^indΔIEC^), tamoxifen (Sigma-Aldrich) was emulsified in corn oil at a concentration of 20 mg/ml 24 and injected daily intraperitoneally at 100 μl per adult mouse (8-12 weeks old) for 5 consecutive days. Littermates carrying the loxP-flanked alleles but not expressing CreERT2 recombinase (hereafter PRMT1^fl/fl^) were injected with the same dose of tamoxifen as the control mice. The mice were euthanized at the indicated time points after the first tamoxifen injection (Fig. 3A). All animal care and treatments were done as approved by the Animal Use and Care Committee of Eunice Kennedy Shriver National Institute of Child Health and Human Development (NICHD), National Institutes of Health (NIH).

### Genotyping

Mouse tail tips were used to isolate genomic DNA with QuickExtract DNA extraction solution (EPICENTRE Biotechnologies). The DNA was used for PCR genotyping with primers for PRMT1 and Cre 23, or with the forward primer 5'-CCAGTTTCCCTTCTTCCTCTG-3' and reverse primer 5'-CGGTTATTCAACTTGCACCA-3'; forward primer 5'-AGTGGCCTCTTCCAGAAATG-3' and reverse primer 5'-TGCGACTGTGTCTGATTTCC-3' for CreERT2. The PCR products were analyzed by 2% agarose gel electrophoresis to determine the genotype based on the sizes of the PCR products.

### Measurements of the small intestine, intestinal crypts, and villi

The length of the dissected small intestine was measured from the beginning of the duodenum to the end of the ileum. The length of the large intestine (colon) was measured from the beginning of the cecum to the end of the rectum. The length of the villus was measured from the mouth of the crypt to the tip of the villus and the length of the crypt was measured from the base to the mouth of the crypt on hematoxylin and eosin (H&E)-stained intestinal cross-sections.

### Histological processing and staining

Isolated intestine was flushed with ice-cold 1X phosphate-buffered saline (PBS) and fixed in 10% neutral buffered formalin (Sigma-Aldrich) at room temperature overnight, then transferred into 70% ethanol, processed with a tissue processor (Excelsior AS Tissue Processor; Thermo Fisher Scientific), followed by embedding in paraffin and then cutting into 5 μm sections.

For H&E staining, paraffin sections of the intestine were stained with H&E by following the manufacturer's protocol (Sigma-Aldrich) and analyzed under a bright-field microscope.

For Alcian Blue staining, paraffin sections of the intestine were stained with an Alcian Blue Kit (Abcam) by following the manufacturer's protocol. The blue goblet cells in the crypt and villus were counted visually or analyzed by using Fiji ImageJ to count the positive cells.

### EdU staining

Adult mice were intraperitoneally injected with 5-ethynyl-2´-deoxyuridine (EdU) (1 mg per mouse) two hours before euthanization and visualized with Edu staining on paraffin-embedded sections. Proliferating cells were labeled with EdU staining by using Click-iT^™^ EdU Cell Proliferation Kit for Imaging (Invitrogen, Alexa Fluor^™^ 594 dye). The tissue sections were counter-stained with Hoechst33342 for 30 min at room temperature. Coverslips were mounted in ProLong ^™^ Gold Antifade reagent (Invitrogen). The fluorescent pictures for different colors and different sections were taken under the same settings and then analyzed by using Fiji ImageJ at the same setting for quantification.

### TUNEL assay

Apoptotic cells were detected with the terminal deoxynucleotidyl transferase dUTP nick end labeling (TUNEL) method by using *In Situ* Cell Death Detection Kit (Roche, TMR red). After incubation with TUNEL reaction mixture at 37°C for 1 h, tissue sections were washed in PBS three times and counter-stained with Hoechst33342 for 30 min at room temperature. Coverslips were mounted in ProLong ^™^ Gold Antifade reagent (Invitrogen). The fluorescent pictures for different colors and different sections were taken under the same settings and then analyzed using Fiji ImageJ at the same setting for quantification.

### Immunohistochemistry

For immunofluorescence analyses, paraffin sections (5 μm) were baked at 60°C followed by dewaxing in xylene and rehydration through a series of different concentrations of ethanol. Antigen retrieval was performed by boiling in an antigen retrieval buffer (1 mM Tris-HCl, 1 mM EDTA, and 0.05% Tween-20) for 3 minutes at 125 °C followed by washing the slides three times in 1 × TBST (1 × TBS and 0.05% Tween-20) for 5 minutes each. Blocking was performed for 10 minutes in 10% normal goat serum TBST for 1 hour at room temperature, then slides were incubated with primary antibody (Ki67 from Cell Signaling Technology at 1:500 dilution; Lysozyme from Dako at 1:500 dilution) at 4°C overnight, followed by incubation with appropriate secondary fluorescent-conjugated antibody (Invitrogen) and DAPI for 1 hour at room temperature. Coverslips were mounted in ProLong ^™^ Gold Antifade reagent (Invitrogen).

### *In situ* hybridization

*In situ* hybridization with RNAscope 2.5 HD Reagent Kit-Brown (322300; Advanced Cell Diagnostics) was performed on 5 μm formalin-fixed, paraffin-embedded sections according to the manufacturer's instructions. The RNAscope probes used were PRMT1 (cat no. 522101), and Lgr5 (cat no. 312171), the positive control probe Ppib (cat no. 313911), and the negative control probe DapB (cat no. 310043).

### Isolation of mouse intestinal crypts

Small intestine and colon were isolated and flushed with cold PBS. The tissues were cut open longitudinally and cut into small fragments roughly 2-4 mm in length. The intestinal fragments were washed twice with cold PBS and then incubated with 20 mM EDTA-PBS on ice for 40 min. Tissue pieces were shaken vigorously, supernatant collected and filtered through a 70-μm filter as fraction 1 in a new conical tube, and the remaining tissue pieces were resuspended in 25 ml PBS containing 0.1% BSA. The process above was repeated and the new supernatant was collected as fractions 2. This was repeated until fraction 6 was collected. The fractions 2-6 typically consisted almost entirely of crypts (viewed under a light microscope). Fractions 2-6 were centrifuged at 300 g for 3 min and the pellets were washed twice in PBS. Cell pellets were either resuspended in TRIzol™ Reagent (Invitrogen) for RNA isolation, or in M-PER™ Mammalian Protein Extraction Reagent (Thermo Scientific) with Complete Protease Inhibitor Cocktail (Roche) and Halt Phosphatase Inhibitor Cocktail (Thermo Scientific) for protein isolation.

### RNA isolation and real-time qPCR

RNA from isolated crypts was extracted by using Direct-zol™ RNA Miniprep (ZYMO Research), 1μg RNA was reverse-transcribed into cDNA by using the High-Capacity cDNA Reverse-Transcription Kit (Applied Biosystems). The qRT-PCR was performed by using SYBR Green PCR Master mix (Applied Biosystems) in a total volume of 10μl with the Step One Plus Real-Time PCR System (Applied Biosystems) with the forward primer: 5'-CACCCTCACATACCGCAACT-3' and the reverse primer: 5'-ATCCCCAATAACCTTGCGGG-3' for PRMT1 (targeting exons 4 and 5); the forward primer: 5'-ATGCCCACCTCCTCAAAGAC-3' and the reverse primer: 5´-GTAGTTTCCGTTGGAACAGTGAA-3' for Muc2. β-actin was used as the internal control with the forward primer: 5'-GGCTGTATTCCCCTCCATCG-3' and reverse primer: 5'-CCAGTTGGTAACAATGCCATGT-3'.

### Protein isolation and Western blot analysis

For protein isolation, the intestinal crypts in the lysis buffer above were homogenized and incubated for 10 min at 4°C, centrifuged (14 000 rpm, 4°C, 15 min) and the supernatant was collected. For Western blot analysis, 10 μg proteins were electrophoresed on a 10%-20% Tris-Glycine gel (Thermo Scientific) and transferred to polyvinylidene fluoride (PVDF) membranes (Immuno-Blot PVDF, BioRad) with TransBlot (BioRad) for 10 min. The membranes were blocked with 4% BSA in TBS-0.1%Tween 20 and then incubated with primary antibodies, rabbit anti-PRMT1 (Upstate, 1:1000 dilution), H4R3me2a (Active Motif, 1:1000 dilution), or mouse β-actin (R&D, 1:2000 dilution) as a loading control at 4°C overnight, followed by incubation with Goat anti-Rabbit IRDye 680® or Goat anti-mouse IRDye® 800 secondary antibody (Odyssey) for 1 hour at room temperature. The proteins of interest were detected by using Odyssey® infrared imaging system (Li-Cor Bioscience), and protein bands were quantified by using Li-Cor software.

### Statistical analysis

Statistical significance of the differences between samples was determined by using a two-tailed unpaired Student's t-test. All experiments were repeated for at least two times. For the analysis of intestinal cross-sections, individual cross-sections instead of individual animals were used as samples for the Student's t-test. All data were expressed as the mean ± the standard error of the mean. Prism 9 from GraphPad software was used to calculate P values and plot figures. *p < 0.05, **p < 0.01, ***p < 0.001. ns no significant.

## Supplementary Material

Supplementary figures.

## Abbreviations

PRMT1: protein arginine methyltransferase 1
ISCs: intestinal stem cells
TA: transit-amplifying
H4R3me2a: asymmetrical dimethylation on arginine 3 of histone H4
IEC: intestinal epithelium cell
SI: small intestine
TM: tamoxifen
RT-qPCR: real-time quantitative PCR
