# Supplementary material for: Protein arginine methyltransferase 1 is required for the maintenance of adult small intestinal and colonic epithelial cell homeostasis
Source: Int J Biol Sci. 2024 Jan 1;20(2):554–68. doi: 10.7150/ijbs.89958 (PMC10758107; doi:10.7150/ijbs.89958)
Supplement: Supplementary file 1 — Supplementary figures. [file ijbsv20p0554s1.pdf]

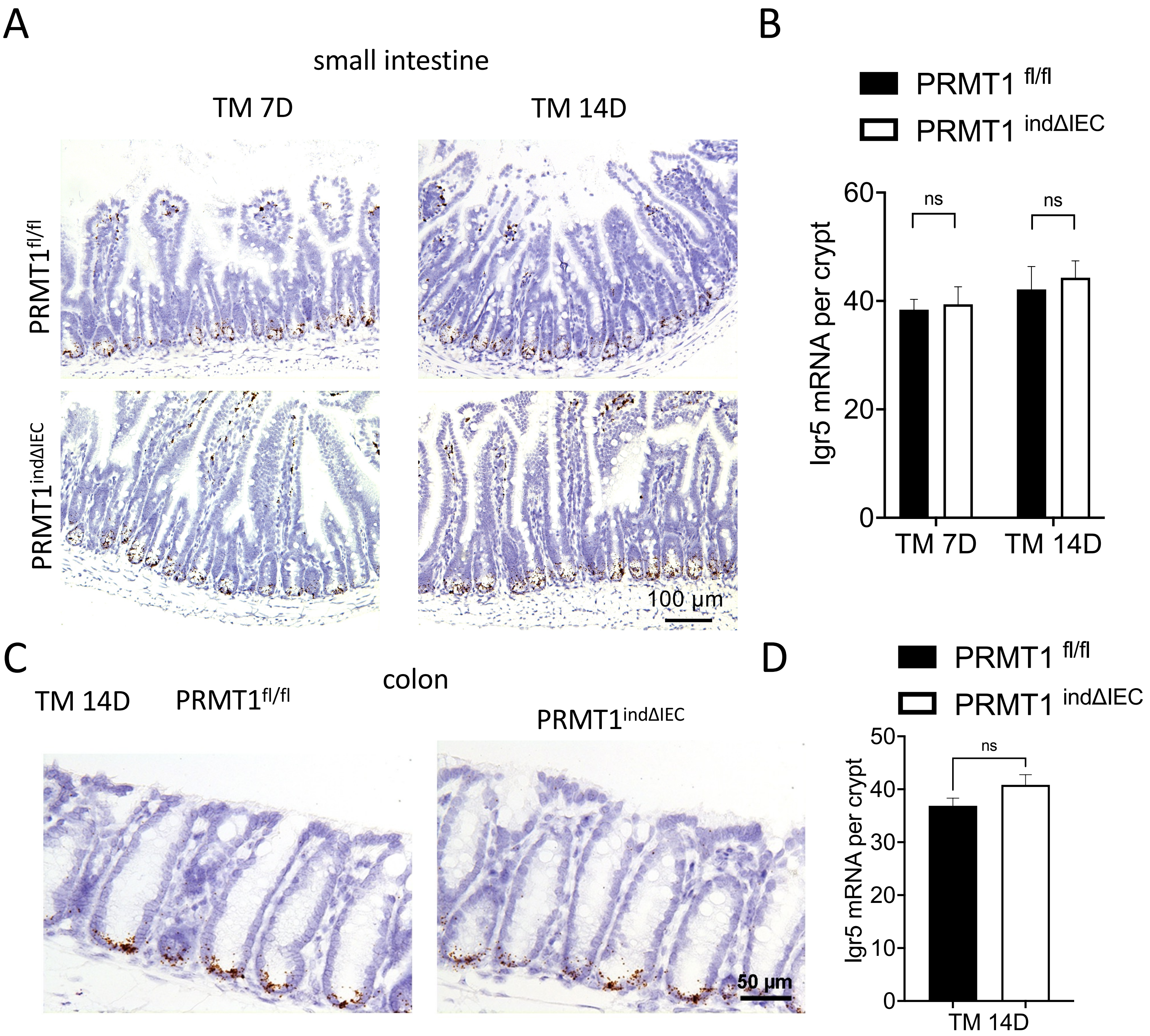

Fig. S1. Inducible PRMT1 deletion in adult intestinal epithelium does not affect intestinal stem cell number.

(A, C) In situ hybridization for Lgr5 on small intestinal (A) and colonic (C) sections from PRMT1$^{ind\Delta IEC}$ and control littermates at indicated days after the onset of TM treatment (See Fig. 3). Note that Lgr5+ cells were located at the base of crypts in the small intestine and colon.

(B, D) Quantitative analyses of the lgr5 expression in the crypt of small intestine (B) and colon (D) were performed on multiple sections per animal for each group. The values were presented as mean ± SEM with n=3-4 mice per group at each time point. ns no significant. Scale bars indicate 100 µm (A), 50 µm (C).

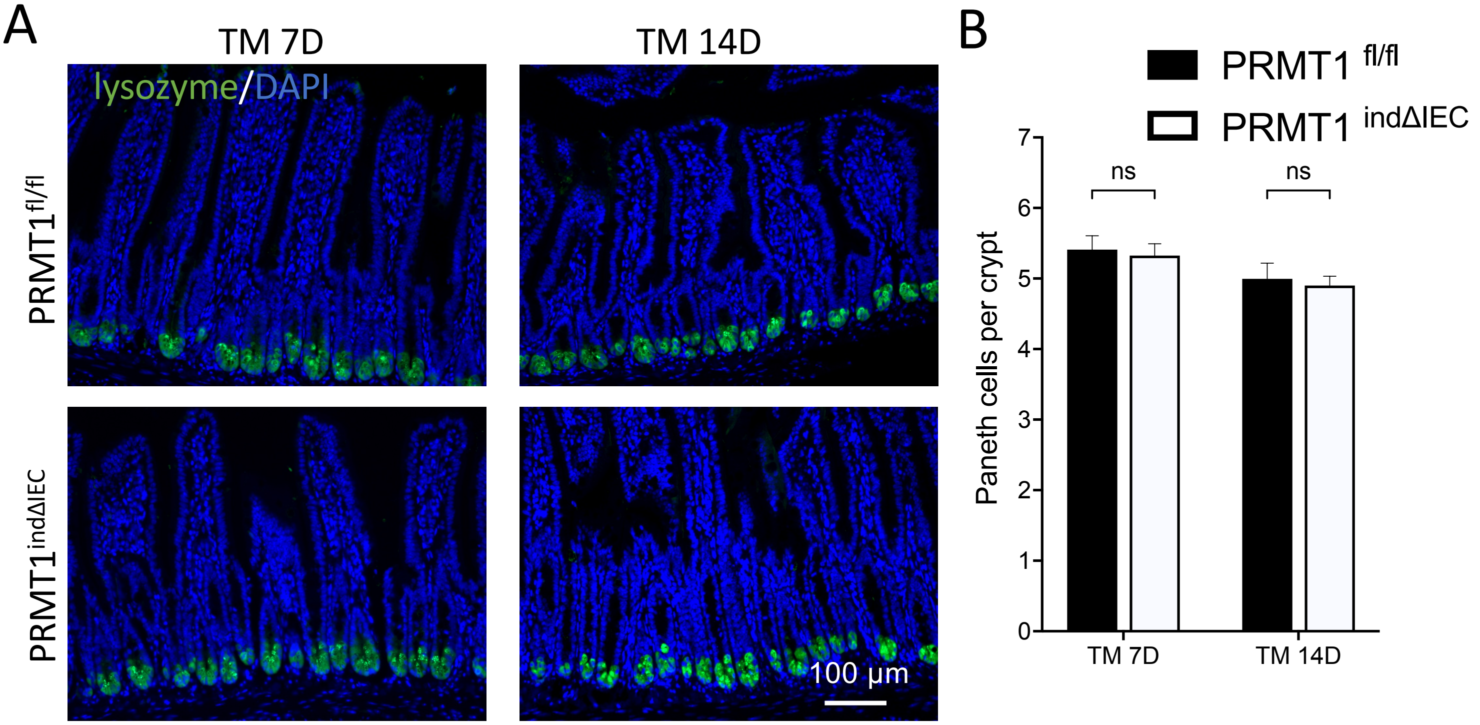

Fig. S2. Inducible PRMT1 deletion in adult intestinal epithelium does not change Paneth cell number in the small intestine.

(A) Immunofluorescent staining for lysozyme in small intestinal sections from PRMT1$^{ind\Delta IEC}$ and control littermates at indicated days after the onset of TM treatment (See Fig. 3). The lysozyme-labeling stained Paneth cells green with the DNA stained purple with Hoechst.

(B) Quantitative analysis of the lysozyme+ cells shows that PRMT1 deletion does not alter the number of Paneth cells. Multiple sections per animal were analyzed for each group. The values were presented as mean ± SEM with n=3-4 mice per group at each time point. ns no significant. Scale bars indicate 100 µm.
